# First description of the male and redescription of the female of *Parahiranetis salgadoi* Gil-Santana (Hemiptera, Reduviidae, Harpactorinae)

**DOI:** 10.3897/zookeys.671.11985

**Published:** 2017-04-26

**Authors:** Hélcio R. Gil-Santana, Adriana Trevizoli Salomão, Jader de Oliveira

**Affiliations:** 1 Laboratório de Diptera, Instituto Oswaldo Cruz, Av. Brasil, 4365, 21040-360, Rio de Janeiro, RJ, Brazil; 2 Programa de Pós-graduação em Ecologia, Instituto de Biologia, Universidade Estadual de Campinas, CP 6109, 13083-970, Campinas, SP, Brazil; 3 Laboratório de Parasitologia, Universidade Estadual Paulista “Julio de Mesquita Filho”, Faculdade de Ciências Farmacêuticas UNESP/FCFAR, Rodovia Araraquara Jaú, KM 1, 14801-902, Araraquara, SP, Brazil

**Keywords:** *Graptocleptes*, Harpactorini, Heteroptera, *Hiranetis*, Neotropics, sexual dimorphism, wasp mimicry

## Abstract

The male of *Parahiranetis salgadoi* Gil-Santana, 2015 is described for the first time, with a redescription of the female of this species based on additional specimens. Comments on possible mimicry and crypsis exhibited by adults and nymphs of this species, respectively, are provided.

## Introduction

Harpactorinae is the largest subfamily of Reduviidae and is represented by the tribes Apiomerini and Harpactorini in the Neotropical region (Gil-Santana et al. 2015). Harpactorini is the most diversified Reduviidae group with more than 53 recognized genera in the Neotropical region (McPherson and Ahmad 2011, Forero 2011, 2012, Swanson 2012, Gil-Santana 2015, Gil-Santana et al. 2015). Several taxa of Harpactorini are thought to be involved in mimicry systems with Hymenoptera (Champion 1899, Elkins 1969, Maldonado and Lozada 1992, Hogue 1993, Gil-Santana 2008, Forero and Giraldo-Echeverry 2015, Gil-Santana 2015, 2016). They resemble bees or wasps in general body and wing coloration as well as physical proportions (Haviland 1931, Leathers and Sharkey 2003, Gil-Santana 2008, 2015, 2016). The striking resemblance resulting from this mimicry has frequently led to the confounding of some closely related genera, such as *Hiranetis* Spinola, 1840 and *Graptocleptes* Stål, 1866 (Gil-Santana et al. 2013).

Sexual dimorphism has been recorded in several species of Harpactorini. In a number of species belonging to *Zelus* Fabricius, 1803, for example, males and females differ drastically in size, body configuration, and coloration (Zhang et al. 2016). In addition to the bigger size and larger abdomen of females, males in several genera have larger eyes and/or a basally-thickened third antennal segment. The latter has been considered to be among the diagnostic features at generic level (Stål 1872, Champion 1899, Martin-Park et al. 2012, Gil-Santana et al. 2013, Gil-Santana 2016). However, sexual dimorphism may also be limited to minor differences in coloration and size, as in many species of *Zelus* (Zhang et al. 2016).

Male genitalia have been found to provide useful diagnostic characteristics for distinguishing species within the genera of Harpactorini (e.g. Elkins 1954a,b, Hart 1975, 1986, 1987, Forero et al. 2008, Zhang et al. 2016). The parameres, medial process of the pygophore, dorsal phallothecal plates, and the struts were the main structures with attributes important at the specific level in studies of *Aristathlus* Bergroth, 1913 (Forero et al. 2008), *Atopozelus* Elkins, 1954 (Elkins 1954a), *Atrachelus* Amyot & Serville, 1843 (Elkins 1954b), *Ischnoclopius* Stål, 1868 (Hart 1975) and *Zelus* (Hart 1986, 1987, Zhang et al. 2016). The endosoma contents, such as its processes, were not examined or recorded in most of these studies. Nevertheless, they have been described for one species of *Graptocleptes* (*G. bicolor* (Burmeister, 1838); Gil-Santana et al. 2013) and one of *Hiranetis* (*H. atra* Stål, 1872; Gil-Santana 2016). These genera are considered to be closely related to each other (Stål 1872, Champion 1899) and to *Parahiranetis* Gil-Santana, 2015 (Gil-Santana 2015, 2016), allowing for important comparisons of several diagnostic traits of male genitalia.

*Parahiranetis* is a monotypic genus containing the wasp-mimicking species *P. salgadoi* Gil-Santana, 2015. This genus was described based on two female specimens collected in the middle of last century (holotype) and in its first half (paratype) in Rio de Janeiro, Brazil (Gil-Santana 2015). Following the publication of the species, the second author (ATS) informed the first author (HRG-S) that she had observed and collected specimens of a wasp-mimicking reduviid in São Paulo, a state neighboring Rio de Janeiro, the type-locality. Surprisingly, the species that she observed was *P. salgadoi*. Through this opportunity, we were able to obtain both males and females, which allowed us to provide the first description of the male and a redescription of the female of this species and also record the morphological variability occurring in this species. We also provide photographs of live immature and adult specimens. The apparent mimicry and crypsis shown by the adults and nymphs, respectively, are also discussed.

## Materials and methods

The specimens described here are deposited in the Entomological Collection of the National Museum of the Federal University of Rio de Janeiro (Museu Nacional da Universidade Federal do Rio de Janeiro), Rio de Janeiro, Brazil (**MNRJ**). When citing the text on the labels of a pinned specimen, a slash (/) separates the lines and a double slash (//) different labels. All measurements are in millimeters (mm).

All fieldwork, including observation and collection of the living specimens from the state of São Paulo, was undertaken by the second author (ATS), who also pinned and dried these specimens for subsequent study. She obtained images of live specimens (Figs 43, 59–63) using a Canon EOS 60D digital camera with a Canon EF 100 mm macro lens.

Scanning electron microscopy images (Figs 4–5, 14–16, 18–21, 46–47, 54, 57–58) were obtained by the third author (JO). The male and female adults and adult female external genitalia from *P. salgadoi* were cleaned in an ultrasound machine. Subsequently, the samples were dehydrated in alcohol, dried in an incubator at 45 ºC for 20 min, and fixed in small aluminum cylinders with transparent glaze. Sputtering metallization was then performed on the samples for 2 minutes at 10 mA in an Edwards sputter coater. After this process, the samples were studied and photographed using a Topcon SM-300 scanning electron microscope, as described by Rosa et al. (2010, 2014).

The photo of the female holotype of *Isthmiade braconides* (Perty, 1832) (Coleoptera: Cerambycidae: Cerambycinae: Rhinotragini) (Fig. 64), which is deposited in the Bavarian State Collection of Zoology (Zoologische Staatssammlung München, ZSM), Munich, Germany, was kindly provided by Dr Steve Lingafelter (Arizona, USA).

All remaining figures were produced by the first author (HRG-S). The fixed adults, microscopic preparations and genitalia were photographed using digital cameras (Nikon D5200 with a Nikon Macro Lens 105 mm, Sony DSC-W830 and Sony DSC-HX400V). Drawings were made using a *camera lucida*. For clarity, the vestiture (setation) was omitted in the ink drawings of Figs 23 and 24. Images were edited using Adobe Photoshop 7.0.

Observations were made using a stereoscope microscope (Zeiss Stemi) and a compound microscope (Leica CME). Measurements were made using a micrometer eyepiece. The total length of the head was measured excluding the neck, for better uniformity of this measurement. Antennal segments were cleared in 20% NaOH solution for 72 hours for microscopic examination (Figs 6–10, 12–13, 49–52). Dissections of the male genitalia were made by first removing the pygophore from the abdomen with a pair of forceps and then clearing it in 20% NaOH solution for 24 hours. Following this procedure, the phallus was found to have spontaneously and completely inflated in one male (Figs 26–29). In another three specimens, the phallus was almost completely everted by carefully pulling on the dorsal endosomal processes using a pair of fine forceps (Figs 30–33). The dissected structures were studied and photographed in glycerol.

General morphological terminology mainly follows Schuh and Slater (1995). Currently, there is a lack of consensus about the terminology to be applied to female and male genitalia in Reduviidae (e.g. Rédei and Tsai 2011). Therefore, in order to maintain uniformity with previous works about species of Harpactorini, the terminology of the male and female genitalia structures follows Davis (1969), Forero et al. (2008), Gil-Santana et al. (2013) and Gil-Santana (2016).

## Taxonomy

### Subfamily Harpactorinae

#### Tribe Harpactorini

##### Parahiranetis salgadoi

Taxon classificationAnimaliaHemipteraReduviidae

Gil-Santana, 2015

Figures 1–4 , 5–13 , 14–16 , 17–20 , 21–27 , 28–32 , 33–36 , 37–41 , 42–45 , 46–52 , 53–58 , 59–62 , 63

Parahiranetis salgadoi Gil-Santana, 2015: 32 [description], 33 [Figures 1–4], 34 [Figures 5–7], 34–35 [description]; Gil-Santana 2016: 93 [citation].

###### Material examined.

*Parahiranetis salgadoi*. **Type material. BRAZIL**, Rio de Janeiro, Rio de Janeiro Municipality, **Holotype** (female): Floresta da Tijuca [Tijuca Forest] / D[istrito] Federal [currently, Rio de Janeiro] / 31.x.1951 / C. A. Campos Seabra [leg.] // [ex] Coleção [Collection] Campos Seabra // ‘XXXII’ // Holotipo [red label] // *Parahiranetis salgadoi* Gil-Santana, 2015 / Gil-Santana det., (MNRJ); **Paratype** (female): Rio de Janeiro / 20.ix.[19]36 / H. S. Lopes [leg.] // Paratipo [red label] // *Parahiranetis salgadoi* Gil-Santana, 2015 / Gil-Santana det., (MNRJ).

###### Additional specimens.

**BRAZIL**, São Paulo, Jundiaí, Serra do Japi, 21.x.2014, 23°13'S, 46°56'W, 1.013 m.a.s.l., 01 female, 01 male, 30.x.2014, 23°14'S, 46°55'W, 900 m.a.s.l., 01 male, 31.x.2014, 23°14'S, 46°55'W, 900 m.a.s.l., 02 males, 03.xi.2014, 23°14'S, 46°55'W, 900 m.a.s.l., 03 females, 01 male, 14.xi.2014, 23°14'S, 46°55'W, 900 m.a.s.l., 01 female, 01 male, 21.xi.2014, 23°13'S, 46°56'W, 1.013 m.a.s.l., 02 males, 1.014 m.a.s.l., 02 females, 01 male, A.T. Salomão leg., (MNRJ).

###### Description.

Male. Measurements are given in Table 1.

**Table 1.** Measurements (mm) of male specimens (N=9) of *P. salgadoi* from São Paulo State.

Measurement	Mean	SD	Maximum	Minimum
Body length to tip of hemelytra	18.0	0.82	19.5	17.0
Body length to tip of abdomen	13.75	0.53	14.7	13.2
Head length (excluding neck)	2.26	0.04	2.3	2.2
Anteocular portion length	0.93	0.04	1.0	0.85
Postocular portion length	0.71	0.03	0.75	0.65
Head width across eyes	1.7	0.05	1.8	1.62
Interocular distance	0.85	0.06	0.9	0.71
Transverse width of eye	0.39	0.01	0.4	0.38
Length of eye	0.61	0.02	0.67	0.60
Antennal segment I length	5.48	0.12	5.7	5.3
Antennal segment II length	1.66	0.06	1.7	1.5
Ant. segment III length **(n = 6)**	9.25	0.35	9.7	8.9
Ant. segment IV length **(n = 3)**	2.03	0.15	2.2	1.9
Max. width ant. seg. III **(n = 6)**	0.26	0.02	0.32	0.24
Labial segment II length	1.33	0.08	1.5	1.25
Labial segment III length	1.33	0.08	1.5	1.25
Labial segment IV length	0.45	0.05	0.5	0.35
Ocellar tubercle width	0.95	0.05	1.0	0.9
Pronotum length	2.57	0.17	2.95	2.4
Pronotum maximum width	2.95	0.14	3.3	2.85
Scutellum length	0.93	0.03	1.0	0.9
Fore femur length	5.62	0.07	5.7	5.6
Fore tibia length	5.97	0.14	6.25	5.8
Fore tarsus length	0.66	0.009	0.67	0.65
Mid femur length	4.9	0.09	5.0	4.8
Mid tibia length	6.3	0.17	6.5	6.0
Mid tarsus length	0.66	0.009	0.67	0.65
Hind femur length	6.7	0.14	6.9	6.5
Hind tibia length	9.4	0.33	9.8	9.0
Hind tarsus length	0.7	0.02	0.75	0.65
Abdomen length	7.78	0.39	8.5	7.2
Abdomen maximum width	2.3	0.36	2.7	1.8

**Figures 1–4.**
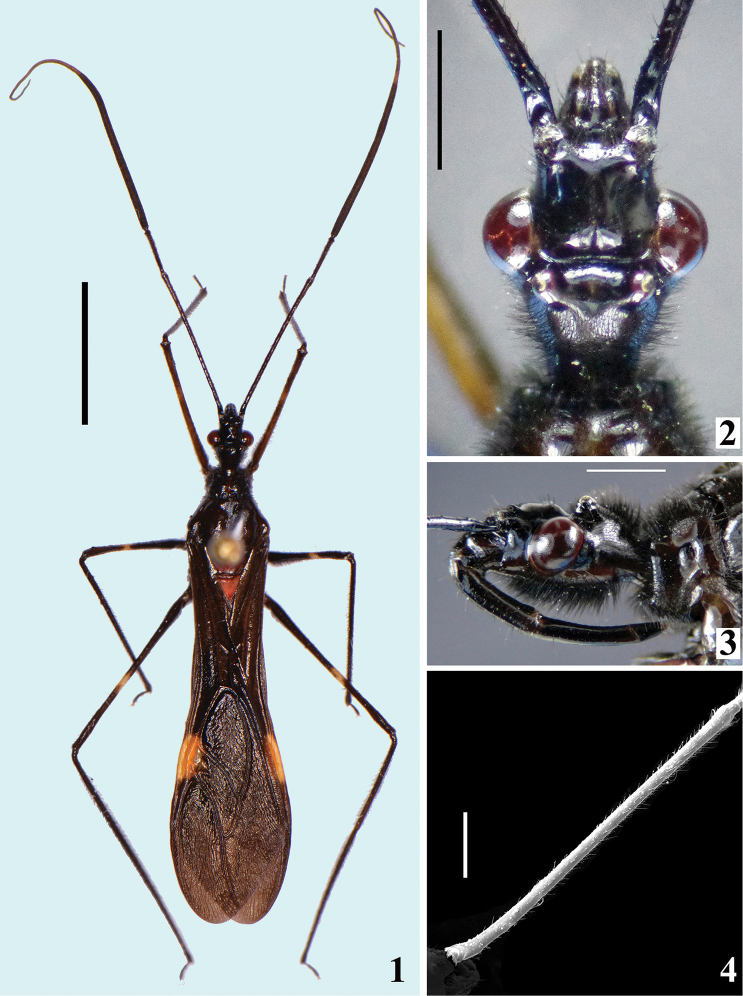
*Parahiranetis salgadoi* Gil-Santana, male. **1** dorsal view **2–3** head **2** dorsal view **3** lateral view **4** antennal segment I, lateral view. Scale bars: 5.0 mm (**1**); 1.0 mm (**2–4**).

*Coloration*: general coloration black, sometimes brownish, with reddish portions (Figs 1–3). Head, including antennae, black (Figs 2–3). Thorax mostly blackish; in some specimens variable blackish-brown on pleura, sterna and ventral portion of coxae; dark-reddish on anterior half of midline on fore lobe of pronotum, including midlongitudinal sulcus, in most specimens; disc of scutellum bright reddish; hemelytra generally black, sometimes dark brown, with a yellowish spot on external and mid-distal portions of corium (Fig. 1), reaching adjacent part of membrane, especially in basal portion of distal cell of membrane (Fig. 17); in one specimen a small dark-reddish spot present at extreme base of hemelytra, adjacent to propleura. Hind wing mostly brownish-black, with clear area at basal portion and in four lines parallel to veins (Fig. 17). Fore femur black to dark brown, frequently with dorsal surface paler, with dark yellowish tinge, and with a yellowish annulus; the latter is as distinct as the annuli on other femora in few specimens, but in most of them, it is very faint, sometimes almost imperceptible, and is situated somewhat distally to midportion of fore femora; this annulus is approximately 1/8 to 1/11 as long as fore femur and its midpoint is about 8 to 11% distal from midpoint of fore femur. Mid and hind femora black, with an evident yellowish annulus situated somewhat distally to midportion of each femur. On mid femora, the annulus is approximately 1/7 to 1/10 as long as the segment and its midpoint is about 5 to 8% distal from midpoint of mid femur, while on hind femora, the same relationships are around 1/8 to 1/11 and 7 to 9%, respectively. In few specimens, the submedian annuli of the mid and hind femora are clearer, almost whitish at midportion of each femur. Tibia and tarsi black; claws clearer, dark-yellowish, sometimes with some reddish tinge. Abdomen: segment II (first visible) almost completely reddish; somewhat elevated transverse anterior area on sternite II is clear, light-yellowish to yellowish; in many specimens, there is a very small linear blackish spot on superior portion of anterior margin of sternite II, just below connexivum. Sternite III mostly reddish, with connexivum completely blackish or mostly blackish with only extreme base or basal half reddish, or only with superior margin darkened; in some specimens area near connexivum is also darkened or blackish to a varying extent. Sternite IV mostly blackish, reddish laterally on anterior half; extent of reddish coloration is variable among specimens; remaining sternites and pygophore almost completely blackish; most specimens with a very small reddish spot on posterolateral angle of last sternite, just below connexivum; other small variations in some specimens: reddish faint tinge on inferior portion of connexivum of sternites IV and/or V; median portion of sternites VI and/or VII brownish-black to a varying extent.

**Figures 5–13.**
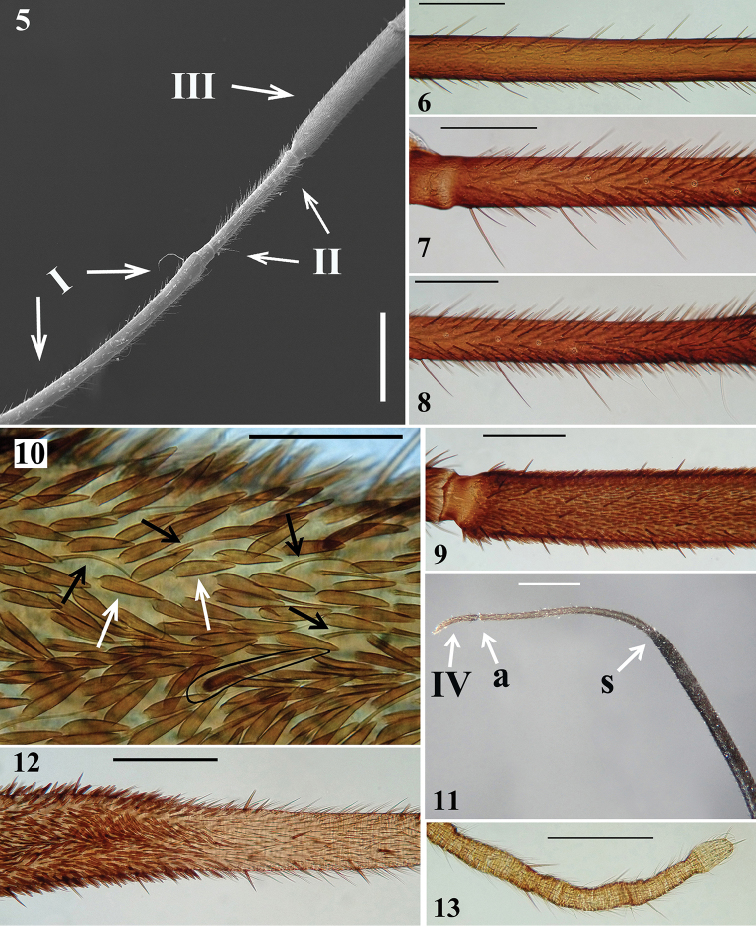
*Parahiranetis salgadoi* Gil-Santana, male antenna. **5** apical half of segment I, segment II and basal portion of segment III **6** segment I, midportion **7–8** segment II **7** basal half **8** distal half **9–10** segment III **9** basal portion **10** small area under higher magnification (a stiff darkened seta contoured; black arrows point to thin pale setae; white arrows point to blackish stiff adpressed setae) **11** segments III (distal half) and IV (basal half). (**s**: point of clear separation between the thickened and thinner portions; **a**: articulation between segment III and IV; IV: fourth segment, partially broken) **12** segment III, region of the transition between the thickened and thinner portions **13** segment IV, distal half. Scale bars: 1.0 mm (**5, 11**); 0.3 mm (**6–9, 12, 13**); 0.1 mm (**10**).

**Figures 14–16.**
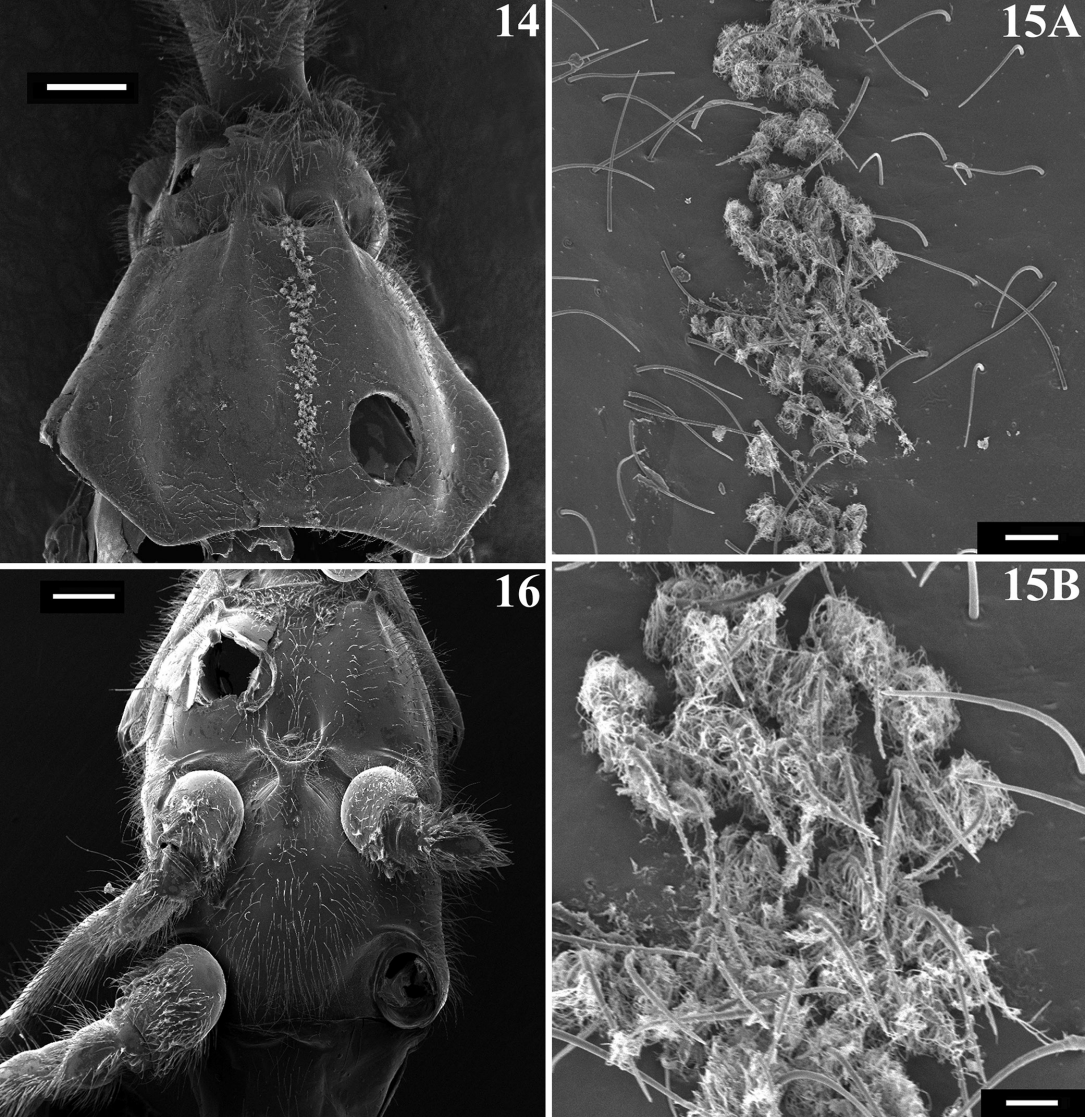
*Parahiranetis salgadoi* Gil-Santana, male thorax. **14** pronotum, dorsal view **15** setae of the midlongitudinal line of the pronotum with flocky patches of wax-like substance **A–B** under progressively higher magnification **16** meso- and metathorax, ventral view. Scale bars: : 0.5 mm (**14, 16**); 0.05 mm (**15A**); 0.02 mm (**15B**).

**Figures 17–20.**
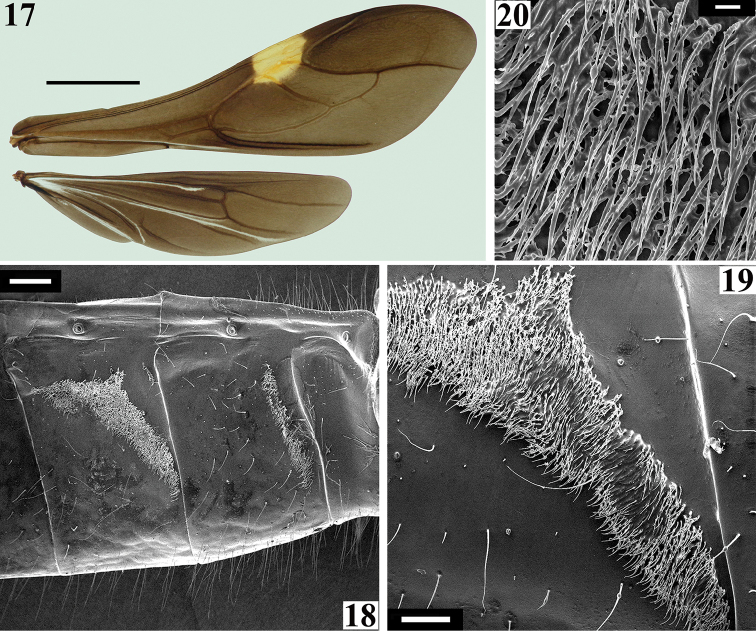
*Parahiranetis salgadoi* Gil-Santana, male. **17** hemelytron and hind wing **18** sternites II–IV, lateral view **19** patch of whitish, minute, thin, adpressed setae on midlateral portion of sternite IV, with few amount of wax-like substance on setae **20** upper portion of the same patch of minute setae on sternite IV under higher magnification. Scale bars: 3.0 mm (**17**); 0.5 mm (**18**); 0.2 mm (**19**); 0.02 mm (**20**).

*Structure and vestiture*: integument mostly shiny, smooth. In some specimens, a thin layer or small patches of sticky substance variably cover integument of antennal segment I and legs, especially on femora and tibiae of fore and mid legs, and/or are scattered on thorax and sternites. Head (Figs 2–3): elongate, approximately 1.3 to 1.4 times as long as width across eyes (length measured excluding neck), almost completely glabrous between eyes; integument shiny, with sparse long and short, straight or somewhat curved blackish setae, which are much denser, forming pubescence of long blackish thick setae on postocular portion and gula. Anteocular portion slightly longer than postocular; the latter, in dorsal view, narrowing gradually to form the neck. Postantennal spines small, somewhat acute or slightly rounded. Interocular sulcus deep, well-marked, curved laterally. A very shallow pair of elevations present anterior to interocular sulcus; sometimes, a thin midlongitudinal furrow present between these elevations. Antenna inserted at level of upper third of eye; antennal segments I and II straight (Figs 1, 4–5); segment I approximately 2.5 times longer than head, with shiny and smooth integument and very sparse, short (with variable lengths), stiff, darkened setae, which become more numerous on mesal surface, approximately in distal two-thirds (Figs 4–6); segments II–IV opaque; segment II, except at glabrous extreme base, covered with numerous short, stiff, semi-erect dark setae, intermixed with similar but longer setae, and a few (about eight to ten) very much thinner isolated elements (interpreted as trichobothria), which are present laterally on basal two-thirds and anteriorly on distal third (Figs 5, 7–8); segment III variably curved (Fig. 1), approximately 1.7 times longer than segment I, conspicuously thickened approximately in basal two-thirds (Figs 1, 5, 9, 11); thickened portion clearly separated in relation to the distal thinner portion (Figs 11–12) and, except at extreme base (which is glabrous) (Fig. 9), completely covered with very short, stiff, blackish, adpressed setae, and with scattered stiff, darkened, semi-erect setae (about twice longer) and a pubescence formed by longer, very thin, pale setae, which are almost imperceptible in this portion (Figs 9–10); distal third of segment III (Fig. 12) and segment IV (Fig. 13) covered with dense pubescence formed by short, thin, pale to whitish setae and with scattered short, darkened, stiff, semi-erect setae; the latter somewhat less numerous on segment IV; segment IV thinnest, moderately curved, with its apex rounded; after drying, segment IV commonly appears to be very distorted with some portions shriveled (Fig. 13). Eyes globose, glabrous, projecting laterally, prominent in dorsal view, reaching dorsal margin of head at interocular sulcus, not reaching ventral margin of head, which is far from inferior margin of the eye (Figs 2–3). Ocelli elevated, much closer to eyes than to each other (Fig. 2). Clypeus straight (Fig. 2). Labium stout, curved, with scattered and somewhat curved, longer and thinner dark setae, reaching prosternum at proximal or mid portion; segment II (ﬁrst apparent) thickest, straight, reaching level of middle portion of eyes, as long as segment III; segment III somewhat curved, reaching or almost reaching level of anterior margin of prosternum; segment IV shortest, triangular, tapering (Fig. 3). Thorax with shiny and mostly smooth integument; prothorax covered with very numerous blackish, thick setae on fore lobe of pronotum and anterior portions of propleura; hind lobe of pronotum with more sparse, shorter and thinner setae on dorsal portion, while on midline thin, short and whitish setae form a midlongitudinal line; basal portion of these setae is frequently covered with rounded, flocky patches of white wax-like substance, enhancing this white stripe (Figs 14–15). Anterior collar inconspicuous; anterolateral angles rounded. Midlongitudinal sulcus on fore lobe of pronotum becoming abruptly deeper at transverse sulcus to form a depression; lateral to the depression, two ill-defined oblique sulci present; transverse sulcus of pronotum not very deep, interrupted before middle by a pair of submedian shallow carina; midlongitudinal furrow on hind lobe very shallow or not evident; lateral longitudinal sulci well marked at posterior half to posterior two-thirds of hind lobe of pronotum (Fig. 14). Humeral angle elevated, rounded at lateral margin (Fig. 14). Scutellum subtriangular, elevated at disc, sometimes somewhat depressed at anterior portion, with scattered, moderately long, dark setae; apex acute, covered with thin, whitish setae, which frequently are basally covered by rounded, flocky patches of white wax-like substance. Posterior portion of propleura, mesopleura, metapleura and thoracic sterna covered with long darkened setae. Mesopleura and metapleura without tubercles. In some specimens, there is a patch of thin whitish setae basally covered with rounded, flocky patches of white wax-like substance, extending from superior margin and posterior portion of mesopleura to anterior portion of metapleura. Mesosternum somewhat elevated laterally, with a median U-shaped carina posteriorly (Fig. 16). Legs: coxae almost to completely glabrous on anterior and basal third portions; fore coxae with scattered, blackish, stout setae posteriorly; mid and hind coxae covered with numerous stout dark setae, including some longer setae on posterior portion and, to a varying extent laterally (Fig. 16). Trochanters densely covered with blackish stout setae and some longer elements ventrally; on ventral surface, one or a pair of fusiform, moderately narrow, glabrous areas. Femora and tibiae slender and elongate. Fore femur slightly longer than or approximately as long as head and pronotum together. All femora slightly dilated subapically, thickened basally, to approximately 1.6 to 1.8 times thicker than narrowest portion of the segment; narrowest portion of femora generally with yellowish annuli (somewhat distally to midportion); apices of all femora with a pair of lateral small tubercles. Femora covered with sparse, long, straight, blackish setae and dense, erect, brush-like setae ventrally, which are longer on basal portion and restricted to basal portion on hind femur. Fore tibiae somewhat enlarged at apex, where there is a dorsal spur and a mesal comb; mid tibiae with uniform thickness; hind tibiae somewhat enlarged in basal half and narrowing a little to apex; all tibiae with scattered, long, thick, blackish setae and dense, shorter, erect, brush-like blackish setae on ventral surface, which become progressively more numerous towards apex; hind tibiae along all portions densely covered with these erect, brush-like blackish setae, which are somewhat longer in slightly enlarged basal half; tarsi with dark shorter setae. Hemelytra long, surpassing abdomen by about half length of membrane; corium covered with curved, adpressed, short dark setae, which are somewhat more numerous over costal and subcostal veins; membrane glabrous; venation of hemelytra and hind wing as in Fig. 17. Abdomen: elongate; spiracles rounded; sternites with integument shiny, covered with long, moderately thin, scattered, dark setae; sternite II with a somewhat elevated transverse area on basal half; fusiform or elongated patches of minute, short, adpressed, thin, whitish setae present on basal half of midlateral portions of sternites III and IV (Figs 18–19); these setae are frequently covered with a variable amount of white wax (Figs 19–20); when more abundant, wax forms flocky patches around and over the setae; when more scarce, wax just covers setae along their lengths (Fig. 20). In some specimens, all abdominal patches of white setae are extensively covered with wax-like substance, while in others, the setae are not covered with the wax or only some of the setae are covered: generally, those of superior part of patches are more extensively covered by wax (Fig. 19).

**Figures 21–27.**
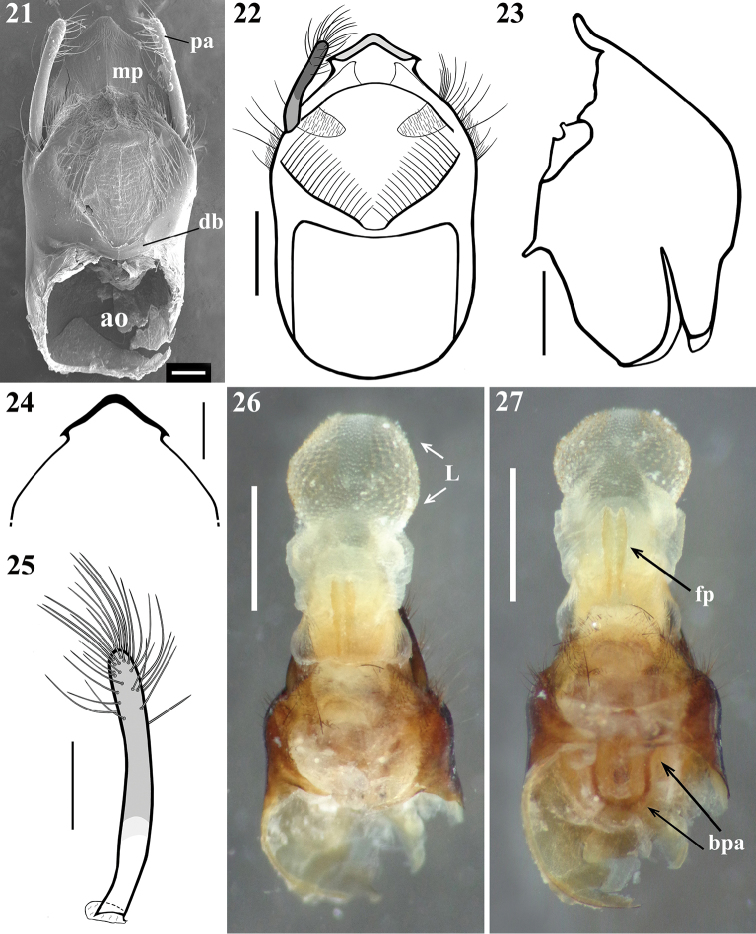
*Parahiranetis salgadoi* Gil-Santana, male genitalia. **21** dorsal view **22** pygophore and right paramere, dorsal view **23** pygophore without parameres, lateral view **24** shape of median process of pygophore, ventral view **25** left paramere **26–27** pygophore with endosoma completely inflated, dorsal view. (**ao**: anterior opening; **bpa**: basal plate arm; **db**: dorsal bridge; **fp**: pair of elongate, flat processes; **L**: globe-shaped lobe in distal third of endosoma; **mp**: medial process of pygophore; **pa**: paramere). Scale bars: 0.2 mm (**21**); 0.5 mm (**22–23**); 0.3 mm (**24–25**); 1.0 mm (**26–27**).

**Figures 28–32.**
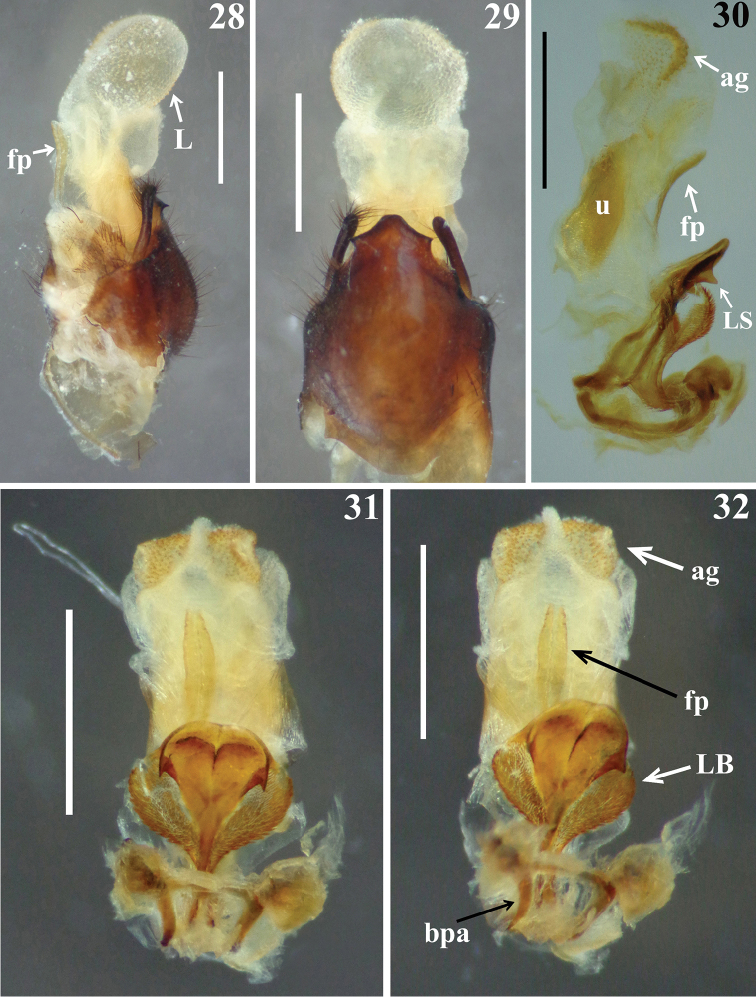
*Parahiranetis salgadoi* Gil-Santana, male genitalia. **28–29** pygophore with endosoma completely inflated **28** latero-dorsal view **29** ventral view **30–32** phallus **30** lateral view **31–32** dorsal view. (**ag**: apicolateral group of spiny processes of the endosoma; **bpa**: basal plate arm; **fp**: pair of elongate, flat processes; **L**: globe-shaped lobe in distal third of endosoma; **LB**: large lobe, middle third of the phallothecal plate; **LS**: lateral large spine of the phallothecal plate; **u**: U-shaped basal process). Scale bar: 1.0 mm.

**Figures 33–36.**
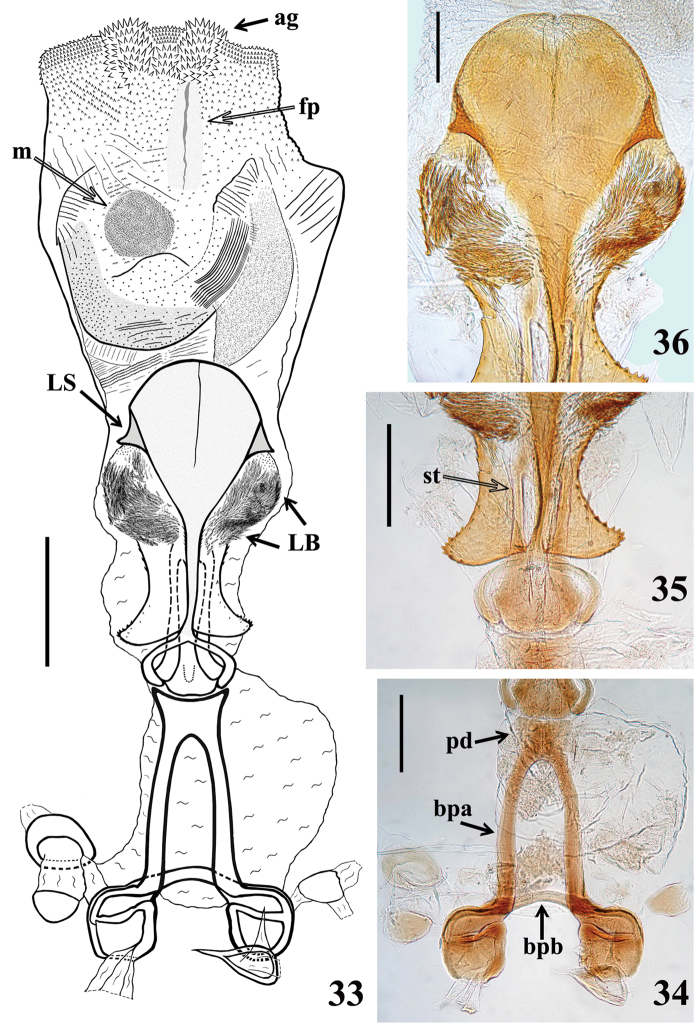
*Parahiranetis salgadoi* Gil-Santana, male genitalia, dorsal view. **33** phallus with expanded endosoma **34** articulatory apparatus **35–36** dorsal phallothecal plate and struts **35** basal portion **36** apical two-thirds. (**ag**: apicolateral group of spiny processes of the endosoma; **bpa**: basal plate arm; **bpb**: basal plate bridge; **fp**: pair of elongate, flat processes; **LB**: large lobe, middle third of the phallothecal plate; **LS**: lateral large spine of the phallothecal plate; **m**: median subspherical process; **pd**: pedicel; **st**: struts). Scale bars: 0.5 mm (**33**); 0.3 mm (**34–35**); 0.2 mm (**36**).

**Figures 37–41.**
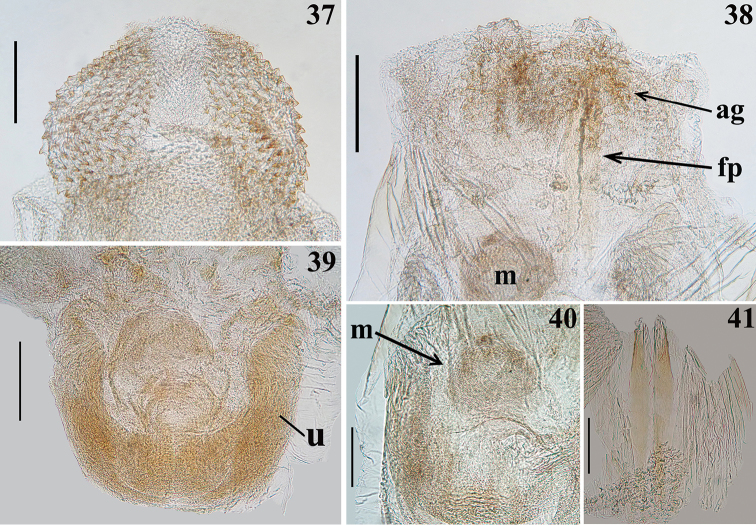
*Parahiranetis salgadoi* Gil-Santana, male genitalia, dorsal view. **37–40** endosoma portions and processes **37** globe-shaped lobe in distal third **38** distal portion **39** basal portion **40** midportion **41** a pair of elongate, flat processes. (**ag**: apicolateral group of spiny processes of the endosoma; **fp**: pair of elongate, flat processes; **m**: median subspherical process; **u**: U-shaped basal process). Scale bars: 0.3 mm (**37–39**); 0.2 mm (**40–41**).

*Male genitalia* (Figs 21–41): pygophore blackish, suboval in ventral view, with an enlarged, somewhat arrow-shaped apex (medial process, mp), in which lateral margins are acutely pointed and the median portion is rounded (Figs 21–22, 24); between anterior and posterior genital openings, a very well sclerotized dorsal (transverse) bridge (db) with a conspicuous median rounded dorsal prominence (Figs 21–22); dorsolateral margin of pygophore (between the bridge and insertions of parameres) with numerous, long, erect setae (Figs 21–22); exposed surface of pygophore with long, thick and dark setae ventrally; these setae are somewhat more numerous on apicolateral portions. Parameres (pa) symmetrical (Fig. 21), rod-like in shape, somewhat curved (Fig. 25); apices rounded, clear at basal third, becoming darker in apical half (blackish); glabrous in basal two-thirds and with long, stout, dark setae in apical third (Figs 21–22, 25); those setae at apicomedial margins even longer (Fig. 25). Phallus elongated, even when not completely inflated (Figs 30–33); articulatory apparatus with long basal plate arms (bpa) (Figs 33–34); basal plate arms (bpa) and basal plate bridge (bpb) narrow and forming a subrectangular set, except in apical portion, where the arms are curved (Figs 33–34); pedicel (pd) moderately short, slightly expanded towards apex (Figs 33–34). Dorsal phallothecal plate weakly sclerotized (Figs 35–36); somewhat expanded laterally at basal third and with small acute spines on lateral margins (Fig. 35); middle third enlarged, with lateral large lobes (LB) covered with very numerous spiny processes (Figs 31–33, 36); distal third together with central portion of basal two-thirds, forming a racket-shaped flat sclerite, with anterior margin largely rounded (Figs 33, 36); its distal third has also a pair of lateral large spines (LS) on its base and a thin median keel, which ends just before the apical margin (Figs 30–33, 36). Struts (st) with subparallel arms (Figs 33, 35–36). Endosoma wall smooth on basal half, becoming progressively more densely, minutely, spiny towards apex (Fig. 33); at distal third, endosoma forms a globe-shaped lobe (L), which is almost completely covered with numerous larger, spiny, somewhat sclerotized processes (Figs 26–29, 37); processes are absent from median portion, where the wall is only minutely spiny (Fig. 37); when the phallus is not completely inflated, larger spiny processes form two apicolateral groups of these processes (ag) (Figs 30–33, 38). The following endosomal processes were observed: 1 - a pair of elongate, parallel, flat, median and weakly sclerotized processes (fp), wrapped in smooth portion of endosoma wall (with fine longitudinal grooves) (Figs 26–28, 30–33, 38, 41); all of these lie dorsally to the other two basal processes described next (Fig. 28); 2 - a larger U-shaped basal process (u) formed by diffuse thickening (Figs 30, 39); 3 - a median subspherical process (m), situated between the lateral arms of the basal process and formed by a dense grouping of small thickenings (Figs 33, 38, 40).

*Female*. Figures 42–58. Similar to male in general. The recorded differences were: size (measurements presented in Tables 2 and 3; see comments below).

*Coloration*: yellowish rings on femora with the following relative values: on fore femora, the annulus is approximately 1/10 to 1/11 as long as fore femur and midpoint of annulus is about 8 to 12% distal from midpoint of fore femur; on mid femora, annulus is approximately 1/7 to 1/8 as long as mid femur and midpoint of annulus is about 8 to 10% distal from midpoint of mid femur, while on hind femora, the same relationships are about 1/7 to 1/11 and 8 to 9%, respectively. On abdominal sternites, generally reddish coloration extends to basolateral portion of sternite V (Figs 55–56).

*Structure*: Head: approximately 1.47 to 1.65 times as long as width across eyes (length measured excluding neck) in the type specimens; labial segment II (ﬁrst apparent) slightly longer than segment III; antennal segment III somewhat thicker in basal portion (Figs 47–48), but much thinner as a whole than that in males, and becoming progressively thinner toward apex, without a clear separation between more or less thickened portions (Fig. 48); segment III is uniformly covered with pubescence formed by thin, pale setae (Fig. 51) (blackish, stiff, adpressed, very short setae that completely cover thicker portion in male are absent); segment III is approximately 1.1 to 1.3 times longer than segment I.

**Table 2.** Measurements (mm) of female specimens (N = 7) of *P. salgadoi* from São Paulo State.

Measurement	Mean	SD	Maximum	Minimum
Body length to tip of hemelytra	22.5	0.64	23.5	21.5
Body length to tip of abdomen	18.45	0.57	19.2	17.5
Head length (excluding neck)	2.6	0.07	2.7	2.5
Anteocular portion length	1.09	0.07	1.2	1.0
Postocular portion length	0.77	0.07	0.9	0.7
Head width across eyes	1.86	0.08	2.0	1.75
Interocular distance	0.98	0.02	1.0	0.95
Transverse width of right eye	0.42	0.04	0.5	0.38
Length of right eye	0.68	0.03	0.75	0.65
Antennal segment I length	6.6	0.18	6.9	6.4
Antennal segment II length	2.42	0.21	2.9	2.3
Ant. segment III length **(n = 4)**	8.02	0.25	8.3	7.7
Ant. segment IV length **(n = 4)**	2.11	0.19	2.3	1.9
Max. width ant. seg. III **(n = 6)**	0.15	0.01	0.17	0.14
Labial segment II length	1.51	0.03	1.6	1.5
Labial segment III length	1.59	0.03	1.65	1.55
Labial segment IV length	0.53	0.04	0.6	0.45
Ocellar tubercle width	1.04	0.07	1.2	1.0
Pronotum length	3.18	0.15	3.4	3.0
Pronotum maximum width	3.77	0.16	4.0	3.5
Scutellum length	1.14	0.11	1.25	1.0
Fore femur length	6.28	0.26	6.7	6.0
Fore tibia length	6.68	0.1	6.8	6.5
Fore tarsus length	0.75	0.0	0.75	0.75
Mid femur length	5.48	0.21	5.9	5.3
Mid tibia length	7.2	0.29	7.5	6.8
Mid tarsus length	0.77	0.02	0.8	0.75
Hind femur length	7.92	0.32	8.5	7.4
Hind tibia length	10.8	0.34	11.5	10.4
Hind tarsus length	0.81	0.03	0.85	0.75
Abdomen length	11.15	0.58	12.0	10.5
Abdomen maximum width	3.7	0.36	4.1	3.1

*External genitalia* (Figs 57–58): syntergite 9/10 (s) with very long, sparse, strong blackish setae; paired gonoplac (g) and posterior margin of ﬁrst gonapophysis (fg) with strong shorter setae.

**Figures 42–45.**
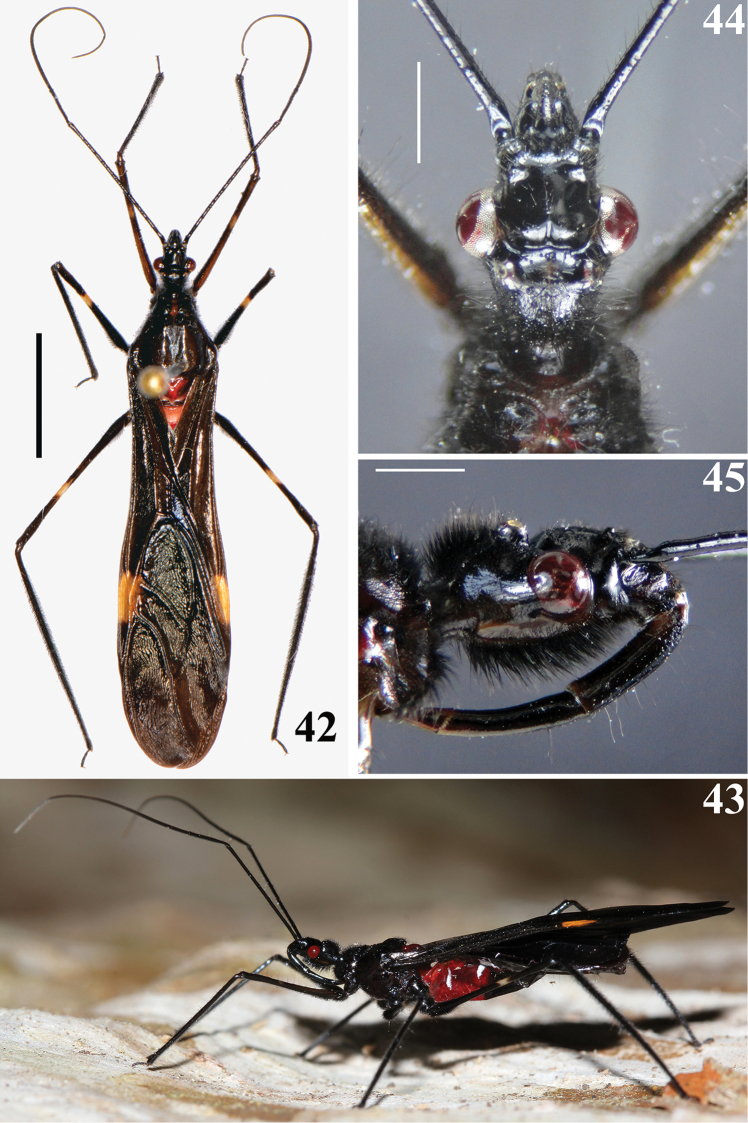
*Parahiranetis salgadoi* Gil-Santana, female. **42** dorsal view **43** live specimen, lateral view **44–45** head **44** dorsal view **45** lateral view. Scale bars: 5.0 mm (**42**); 1.0 mm (**44–45**).

**Figures 46–52.**
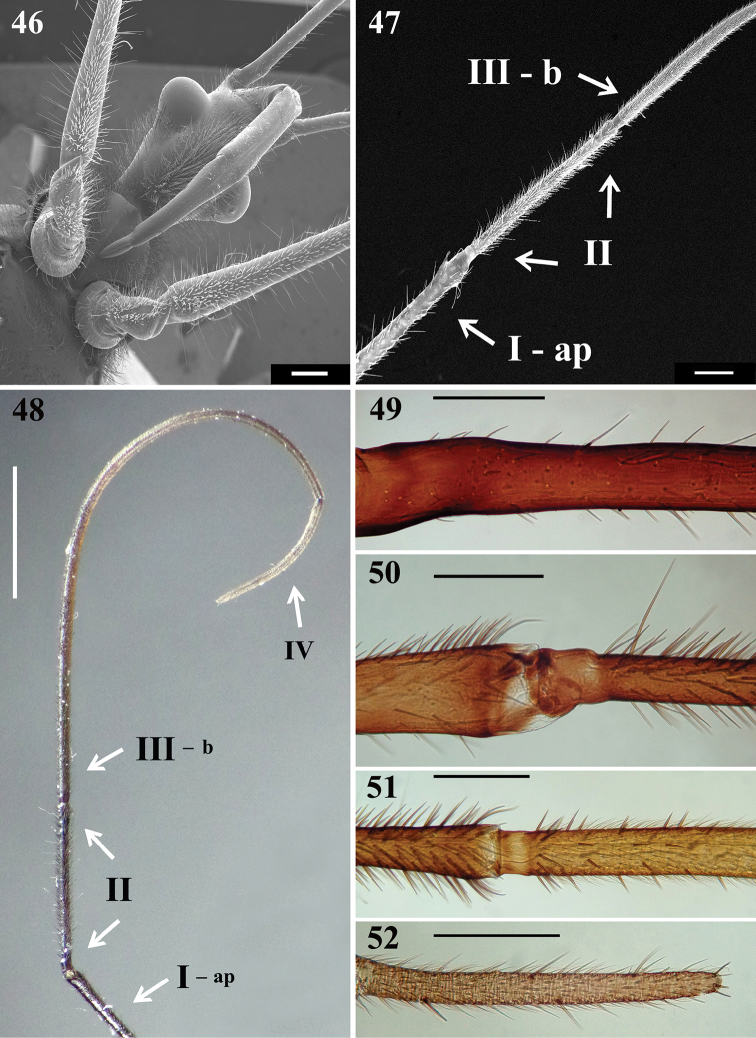
*Parahiranetis salgadoi* Gil-Santana, female. **46** head, fore coxae, fore trochanters and basal half of fore femora, ventral view **47–52** antenna **47** apex of segment I, segment II and basal portion of segment III **48** apex of segment I and segments II–IV. (**ap**: apex; **b**: basal portion) **49** basal portion of segment I **50** apex of segment I and basal portion of segment II **51** apex of segment II and basal portion of segment III **52** apex of segment IV. Scale bars: 0.5 mm (**46–47**); 2.0 mm (**48**); 0.3 mm (**49–52**).

**Figures 53–58.**
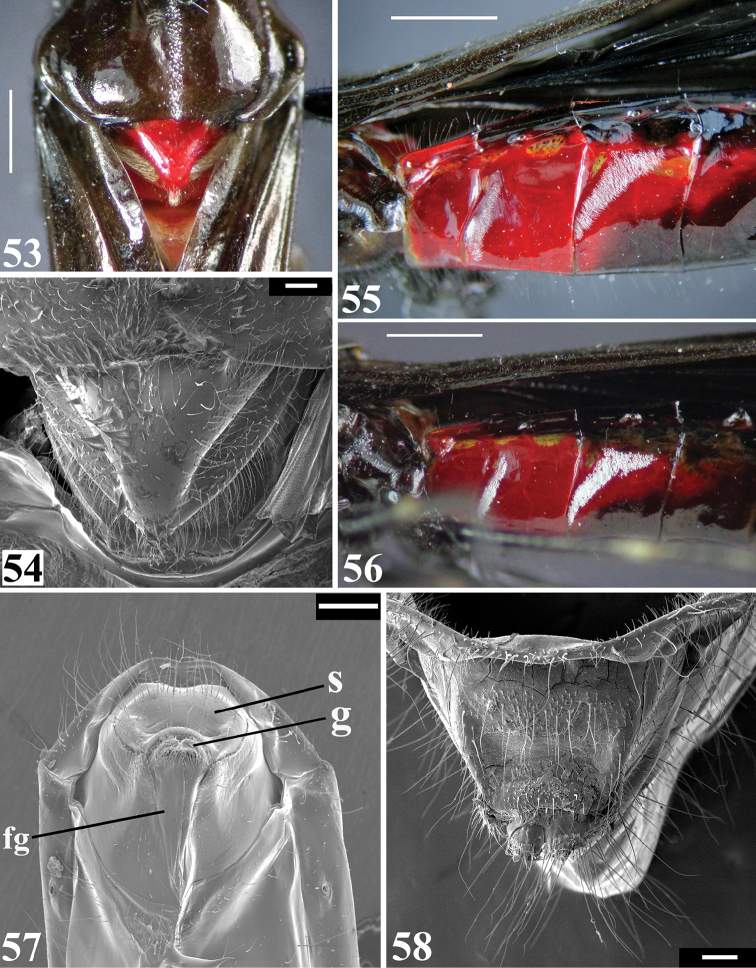
*Parahiranetis salgadoi* Gil-Santana, female. **53–54** dorsal view **53** posterior half of pronotum and scutellum **54** scutellum **55–56** abdominal segments II–IV, lateral view **55** patches of whitish, minute, thin, adpressed setae on midlateral portions of sternite III–IV without wax-like substance **56** the same with large amount of wax-like substance on setae **57–58** female genitalia **57** postero-ventral view (**fg**: ﬁrst gonapophysis; **g**: gonoplac; **s**: syntergite 9/10) **58** posterior view. Scale bars: 1.0 mm (**53**); 0.2 mm (**54, 58**); 2.0 mm (**55–56**); 0.5 mm (**57**).

###### Comments.

In the type specimens, the head and the body length to the tip of the hemelytra are slightly longer than those in the specimens from the state of São Paulo. The body length to the tip of the abdomen is also slightly longer in the holotype (Table 3). Moreover, all females are larger than males (Tables 1–3). The minimum body length in females (to tip of hemelytra/tip of abdomen: 21.5/17.5) is greater than the maximum body length in males (19.5/14.7). Most of the other measurements are proportionally greater in females, in accordance with their bigger size (Tables 1–3). One clear exception is the antennal segment III, which is longer in males (8.9 to 9.7 mm in length; n = 6) than in females (7.7 to 8.3 mm in length; n = 4). Also, the antennal segment III shows evident thickening, approximately in basal two-thirds in males (maximum width: 0.24–0.32 mm) (Figs 1, 5, 9, 11), but not in females (maximum width: 0.14–0.17 mm) (Figs 42, 47–48, 51). This thickened region in males is completely covered by blackish, stiff, adpressed and very short setae (Figs 9–10, 12), which are absent in females (Fig. 51).

**Table 3.** Selected measurements (mm) of female specimens of *P. salgadoi*.

Measurement	holotype	paratype	females from São Paulo State (maximum-minimum)
Body length to tip of hemelytra	24.5	24.2	23.5–21.5
Body length to tip of abdomen	19.5	19.1	19.2–17.5
Head length (excluding neck)	3.3	3.1	2.7–2.5
Head width across eyes	2.0	2.1	2.0–1.75
Interocular distance	1.1	1.0	1.0–0.95
Abdomen length	10.5	11.0	12–10.5
Abdomen maximum width	4.2	4.0	4.1–3.1

###### Distribution.

Brazil, in states of Rio de Janeiro and São Paulo (Atlantic tropical moist forest and Atlantic semideciduous forest, respectively).

###### Observation of live specimens.

**Figures 59–62.**
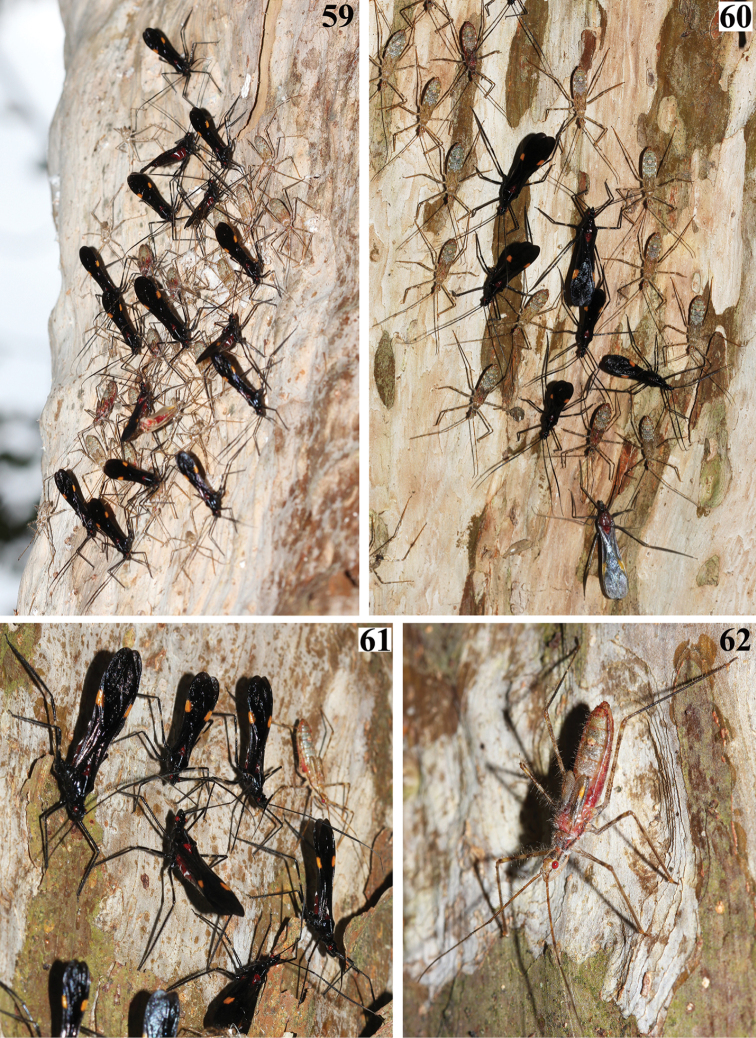
*Parahiranetis salgadoi* Gil-Santana, live specimens. **59–61** aggregates of adults and fifth instar nymphs on tree trunks **62** a fifth instar nymph.

**Figures 63–64.**
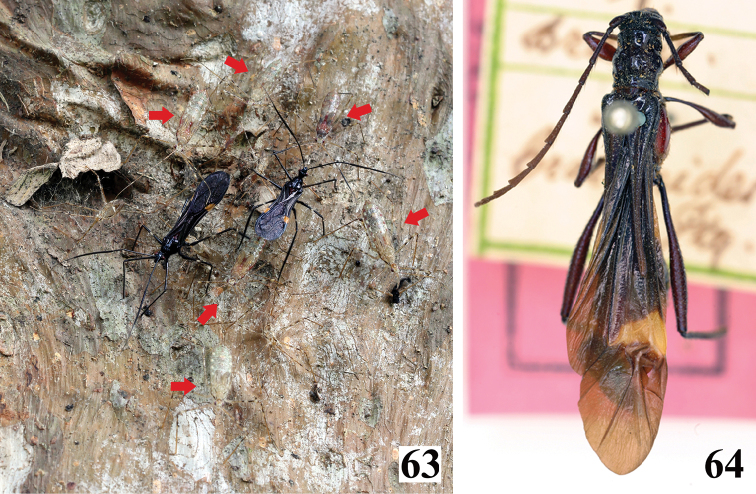
**63** *Parahiranetis salgadoi*, live specimens; two male adults and fifth instar nymphs on a tree trunk; red arrows point to nymphs **64** female holotype of *Isthmiade braconides* (Perty, 1832) (Coleoptera: Cerambycidae: Cerambycinae: Rhinotragini), deposited in the ZSM, Munich, Germany (courtesy of Steve Lingafelter).

Both nymphs and adults were observed in aggregates on tree trunks, where most individuals remained relatively motionless (Figs 59–63). The general coloration of nymphs is quite different from that of the adults. Nymphs have a general color pattern in which shades of gray and light brown predominate (Figs 59–60, 63). However, fifth-instar nymphs just before molting become reddish and progressively darker (Fig. 62).

Nymphs were not collected to allow them to reach the adult stage in the wild. Consequently, it is not possible to provide a formal description of them at this time.

## Discussion

Examination of additional specimens, including males, allowed us to provide a more complete description of the adults of *Parahiranetis salgadoi*. Gil-Santana (2015), for example, had not recorded the sticky substance on some parts of the body and appendages or the white wax-like substance covering groups of setae on the thorax and abdomen (Figs 14–15, 19–20, 53–54, 56), as both were completely absent from the type specimens. Moreover, the sticky substance was present only on some specimens from São Paulo, while the wax-like substance was sometimes absent from portions where it was observed on other specimens (Figs 55–56). Both substances may be lost during manipulation of the individuals and, in the case of the wax-like substance, this may also include loss of the thin fragile setae associated with it (HRG-S pers. obs.). However, the patches of short adpressed setae on the midlateral portions of sternites III and IV were completely covered with wax in some specimens (Fig. 56), while others clearly had no wax and the setae were nonetheless well preserved (Fig. 55). This observation makes it clear that individual differences (at least regarding this trait) can be found even in the same population and at the same observation time, possibly associated to the age and/or behavioral state of each specimen prior collection. Thus, in the type specimens, both substances may have been inadvertently removed; or may have disappeared over time, taking into account the fact that these are old specimens; or alternatively, these specimens may never have had either substance. In any case, it is no longer possible to know the extent to which these substances were present when the specimens were alive.

The sticky, viscous substance that covers the integument of some species of Harpactorini, particularly their fore and mid legs, is produced by specialized epidermal glands. These species use it to catch their prey (Zhang and Weirauch 2013a). Using comparative phylogenetic methods, Zhang and Weirauch (2013b) showed that species of Harpactorini that use a ‘sticky trap predation’ strategy have evolved fore femora that are more slender and longer than those of “non-sticky” bugs. Among some other members of Harpactorini, sticky glands were recorded in species of two genera that are considered close to *Parahiranetis*: *Hiranetis braconiformis* (Burmeister, 1835) and *Graptocleptes* sp. (Zhang and Weirauch 2013a). Interestingly, in the phylogenies obtained by Zhang and Weirauch (2013b), species of the wasp-mimicking genera *Graptocleptes*, *Hiranetis*, *Myocoris, Neotropiconyttus* and *Xystonyttus* were nested in the same clade, among other clades that together make up the “sticky bug clade” within Harpactorini.

Body parts covered with patches of setae with whitish wax-like material have been registered in some Harpactorini species, such as *Cosmoclopius curacavensis* Cobben & Wygodzinsky, 1975 (Cobben and Wygodzinsky 1975), *Harpactor angulosus* (Lepeletier & Serville, 1825) (Pikart et al. 2014), various species of *Heza* Amyot & Serville, 1843 (Maldonado 1976) and *Sphedanolestes zhengi* Zhao, Ren, Wang & Cai, 2015 (Zhao et al. 2015). However, it is clear from the present study that both the sticky and the wax-like substance may be absent when specimens are examined and described, and thus the extent of their existence may remain unknown. The presence or absence of the sticky glands that produce the sticky substance has been shown to correlate with the phylogeny of Harpactorini and faster rates of evolution among “sticky bugs”, in comparison with other reduviids (Zhang and Weirauch 2013b). Similarly, records of the presence or absence of a wax-like substance may turn out to be an additional feature of systematic or taxonomic importance, in the same way as suggested for the “extensive sericeous areas on the abdominal sterna” of *Heza ventralis* Stål, 1872 (Maldonado 1976). Therefore, future studies on Harpactorini should include careful handling of the specimens after collection, to avoid unintentional removal of these substances from their bodies. It is also recommended that this information should be included in the records and/or descriptions whenever present.

Differences in body size and in the structure and vestiture of the third antennal segment were clear-cut enough to be considered as sexually dimorphic in *P. salgadoi*. Despite the small number of measurements obtained for the third antennal segments (because they were broken and lost in some specimens), adults can be sexed readily with the naked eye, by observing these two traits. Firstly, females were larger than males in almost all the morphological characteristics measured. Secondly, the third antennal segment was longer in males than in females, both in absolute and in relative terms (i.e. total length and the ratio between the third and first antennal segments, respectively). Moreover, in males, the third antennal segment was distinctly thickened, approximately in its basal two-thirds, with the thicker portion clearly separated in relation to the thinner, distal portion (Figs 1, 5, 9, 11–12). In females, it showed little or no thickening (Figs 42, 47–48, 51). Two of the sexual differences pointed out in *P. salgadoi* (i.e. females larger than males and the latter with third antennal segment thickened) are concordant with several observations in the literature (Champion 1899, Martin-Park et al. 2012, Gil-Santana et al. 2013, Gil-Santana 2016). Additionally, the thickened portion of the third antennal segment in males was completely covered by very short, stiff, adpressed, blackish setae (Figs 9–10, 12), which were absent in females (Fig. 51). Small differences in coloration were also recorded (see above); however, more specimens need to be examined in order to clarify whether such differences might be due to sexual dimorphism.

Some slight differences were also noted between the type specimens and the specimens from São Paulo. These are very probably due to intraspecific variation. However, measurements of the head and the ratio of head length to maximum width across the eyes are important for separating *Parahiranetis* from related genera, especially *Hiranetis* (Gil-Santana 2015, 2016). Gil-Santana (2015) measured the length of the head including the neck and recorded that it was 1.6 to 1.8 times as long as width across the eyes (in the paratype and holotype, respectively). In the present study, in which head measurements excluded the neck, the head was recorded in the type specimens as being 1.47 to 1.65 times as long as width across the eyes (paratype/holotype). In parallel, this ratio was approximately 1.3 to 1.4 in the specimens from São Paulo. Therefore, the main diagnostic characteristics that separate *Hiranetis*, *Graptocleptes*, and *Parahiranetis* according to Spinola (1840a,b), Stål (1866, 1872), Maldonado and Lozada (1992), Gil-Santana et al. (2013), Gil-Santana (2015, 2016) and this work, are the following:

1) *Graptocleptes*: Head elongate, approximately 1.1 to 1.3 times as long as width across eyes, sparsely setose; legs thicker; fore femur shorter than head and pronotum together and of uniform thickness.

2) *Hiranetis*: Head gibbous, large, approximately as long as width across eyes, densely setose on ventral and post-ocular portions; legs elongated, slender; fore femur subequally longer than head and pronotum together, thicker basally.

3) *Parahiranetis*: Head elongate, approximately 1.3 times to 1.7 as long as width across eyes, densely setose on ventral and post-ocular portions; legs elongated, slender; fore femur subequally longer than head and pronotum together, thicker basally.

The male genitalia of *P. salgadoi* showed some similarities to those of *G. bicolor* (Gil-Santana et al. 2013) and *H. atra* (Gil-Santana 2016), such as: (i) parameres similar in shape and somewhat similar in vestiture; and (ii) presence of a pair of elongate, parallel, flat, weakly sclerotized endosomal processes. However, the latter were recorded with different locations and shapes in each of these species. Comparatively, they are somewhat more elongated, straighter and closer to each other in *P. salgadoi* (Figs 26–28, 31–33, 38, 41). The pygophores of all three species have a somewhat large medial process that is medially rounded at the apex, but in *G. bicolor* and *H. atra* it is subtriangular in shape, while in *P. salgadoi*, it is somewhat arrow-shaped, with the lateral margins acutely pointed (Figs 22, 24, 29). U-shaped and median subspherical endosomal processes that are very similar to those of *P. salgadoi* (Figs 33, 38–40) were recorded in *H. atra*. The distal third of the endosoma of *P. salgadoi*, except in the median portion, is almost completely covered by numerous larger spiny somewhat sclerotized processes (Fig. 37), while in the endosoma of *G. bicolor*, groups of larger similar spiny processes were also recorded. However, in the latter species, these processes were recorded as occurring mostly at the midportion, apically or subapically, and were more sclerotized and clearly triangularly shaped.

Moreover, the male genitalia of *P. salgadoi* differ from those of both of those species in some parts. Firstly, the basal plate arms of the articulatory apparatus are clearly longer in this species (Figs 33–34). Secondly, there is a very remarkable difference concerning the struts and especially the dorsal phallothecal plate. The latter has already been recorded as distinctive between *G. bicolor* and *H. atra*, but the differences are even more accentuated in *P. salgadoi*. The shape of the dorsal phallothecal plate and the portions of it (including the lateral large lobes) that are covered with very numerous spiny processes in the middle third, along with the racket-shaped flat sclerite extending towards the apex with lateral large spines, are striking features and may be unique for this species (Figs 30–33, 35–36). Thus, in agreement with previous studies (Elkins 1954a, b, Hart 1975, 1986, 1987, Forero et al. 2008, Zhang et al. 2016), the features of the male genitalia of *P. salgadoi* that should especially be taken into consideration for comparative purposes are the shape of the medial process of the pygophore and the features of the phallothecal plate.

Historically, only the pattern of yellowish or straw-colored hemelytra with a median transverse black band has received attention in regards to the supposed mimicry between Harpactorini and species of hymenopteran Ichneumonidae and Braconidae (Champion 1899, Haviland 1931, Maldonado and Lozada 1992, Hogue 1993, Leathers and Sharkey 2003, Hespenheide 2010). However, some species that have similar patterns of blackish wings with yellowish ‘pterostigmata’ arise as possible candidates for mimetic complexes, including *P. salgadoi* (Gil-Santana 2015). These are the wasp-mimicking harpactorines *Graptocleptes bicolor*, *G. haematogaster* (Stål, 1860), and an undescribed species of *Hiranetis* as well as the ichneumon wasp *Pedinopelte gravenstii* (Guérin-Méneville, 1826) and the cerambycid beetle *Isthmiade braconides* (Perty, 1832) (Fig. 64). All of these have been recorded from southeastern Brazil, particularly in the states of Rio de Janeiro and/or São Paulo (harpactorines: Stål 1860, 1872, Gil-Santana et al. 2013, Gil-Santana 2015; ichneumon wasp: De Santis 1980; cerambycid beetle: Zikán and Zikán 1944, Zajciw 1972, Monné 1993, Monné and Bezark 2009).

Although there are records of color variation in some wasp-mimicking Harpactorini, at least in the species with the pattern of darkened or blackish hemelytra with yellowish pterostigmata, there is no variation in this pattern. The yellowish pterostigmata is always present (e.g. Gil-Santana et al. 2013, this study). Concerning this point, it is appropriate to correct an assertion by Gil-Santana et al. (2013). In the latter report, it was alleged that Stål (1872) would have “recorded variation of the coloration of the corium of hemelytra of *Graptocleptes bicolor*, describing it as either paler (“Var. b. - *pallidior*”) or completely blackish (“bb. *Corio toto nigricante*”)”. This was a double misinterpretation of Stål’s text. The statement “Var. b. - *pallidior*” did indeed refer to *G. bicolor*, but it was in relation to a “variety” with paler general coloration, and this did not necessarily relate to its hemelytra. Regarding the second assertion, an accurate reading of Stål’s paper makes it clear that “*Corio toto nigricante*” was considered to be a characteristic of another species: *Graptocleptes sanguineiventris* Stål, 1862. Moreover, just above this, Stål (1872) included the previous alternative “b.”, through which the characteristic of “*Corio apice vel prope apicem pallescente vel croceo*” was attributed to another species (*G. gastricus* (Stål, 1860), *G. haematogaster* and *G. bicolor*). This is a clear description of the pale to yellowish spot on the distal portion of the corium of the hemelytra in all these three species. Therefore, the alternative “bb.” (corium completely blackish) was clearly stated as opposite to this latter and was diagnostic of another species (*G. sanguineiventris*).

In contrast to the adults, nymphs of *P. salgadoi* were quite inconspicuous on the tree trunks where they naturally occurred (Figs 59–63). Their cryptic coloration probably has a protective function, which is present to some extent in almost every family of Heteroptera (Schuh and Slater 1995). Ecological studies might improve the current understanding of the contrasting strategies adopted by different life stages in *P. salgadoi*. Additionally, as emphasized by Gil-Santana (2015), it is necessary to elucidate which species or groups of insects sharing the same color pattern as in *P. salgadoi* (i.e. blackish to reddish coloration with yellowish ‘pterostigmata’ on wings and/or yellowish markings on legs) are involved in possible mimicry complexes.

## Supplementary Material

XML Treatment for Parahiranetis salgadoi
